# Predicting the Future With Wearable Technology∗

**DOI:** 10.1016/j.jacasi.2021.10.001

**Published:** 2021-12-21

**Authors:** Alan C. Kwan, Neal Yuan, David Ouyang

**Affiliations:** a Department of Cardiology, Smidt Heart Institute, Cedars-Sinai Medical Center, Los Angeles, California, USA; b School of Medicine, University of California, San Francisco, California, USA; c Section of Cardiology, San Francisco Veterans Affairs Medical Center, San Francisco, California, USA; d Division of Artificial Intelligence in Medicine, Cedars-Sinai Medical Center, Los Angeles, California, USA

**Keywords:** atrial fibrillation, disease screening, wearable technology

**Figure:**
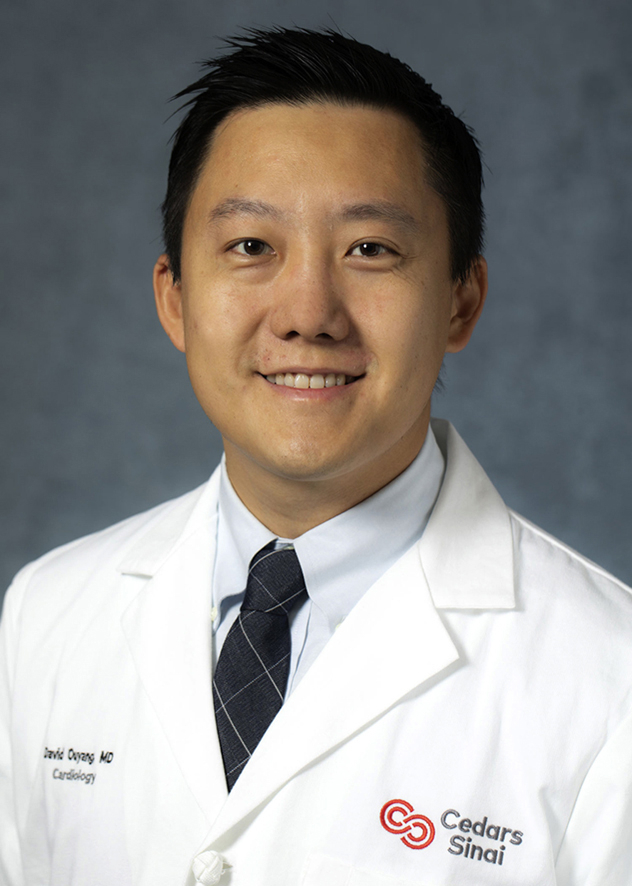

Thinking back to only a decade ago, one remembers when what we now refer to as wearable medical technologies were first marketed as “fitness trackers.” Paralleling Moore’s law, in which complex sensors and computational power get exponentially smaller, modern wearable monitors can pack many more sensors into a small package and track respiratory rate, heart rate, acceleration, and other metrics that previously required uncomfortable chest-straps, many electrocardiography (ECG) leads, and magnetically attached clips. Current technology is now nearly unrecognizable from early pedometers in both form and function, as wearables can sit comfortably on your wrist, display information with slick displays, and collect longitudinal data that provide unparalleled granularity about our daily lives. With the advances in data collected, there has also been a change: These trackers are no longer entirely geared toward “fitness,” but more toward “health,” that is, identifying disease, tracking activity, and providing many other metrics beyond simple exercise. The question has become, where is this technology (and the data) going?

The work by Guo et al (1) in this issue of *JACC: Asia* demonstrates potential next steps for the life cycle of these new technologies in the form of photoplethysmography (PPG)–based heart rhythm monitoring. In the paper, the authors used data from the Huawei Heart Study (2) to predict atrial fibrillation events 4 hours before the onset of the arrhythmia. Building on prior models of atrial fibrillation (AF) detection with the use of PPG, the researchers identified 17 features derivable from the PPG signal that appeared to change extensively when close to the onset of an AF episode. These features were then used in an XGBoost machine-learning model that was trained on data from 273,743 individuals to predict incident AF. Focusing on the 554 individuals with detected AF, this model was then further optimized with the use of feature extension and hyperparameter search, resulting in an optimized model and then tested prospectively for AF detection in a cohort of 50 individuals with paroxysmal AF. In a cohort of participants with the wearable as well as a Holter monitor, the refined model performed well, and predicting AF events 4 hours before onset with an area under the curve (AUC) of 0.826 and positive predictive values (PPV) and negative predictive values (NPV) of 96.4% and 83.1%, respectively.

This study builds on prior work that established the feasibility of using PPG monitoring with wearable devices for real-time AF detection. The Apple Heart Study (3,4) identified AF in patients by detecting heart rate irregularity based on PPG monitoring with an Apple watch. Other studies using different methods and devices, including previous publications from the Huawei Heart Study, have shown similar results. (2,5,6) While those studies demonstrated the ability of PPG technology to detect AF, the present study addresses the more difficult task of predicting future AF before an arrhythmic event has even started. A notable study in this area of AF prediction showed that a deep learning–based approach using 12-lead ECG was able to predict future AF with relatively high fidelity (AUC: 0.87, overall accuracy 83.3%), but the predicted end point was long-term AF, which might be identified by correlates of atrial structure identifiable on ECG (6,7). The present study is one of the first studies to use real-time sensor data for future AF prediction in near real time, potentially providing the opportunity for pill-in-pocket approaches and other novel approaches for paroxysmal disease.

The clinical applications from this research are promising and deserve further exploration. The ability to detect AF onset within a 4-hour window could conceivably provide actionable information that could be used to change an individual’s behaviors (eg, limit alcohol consumption or other AF-triggering activities) or decide on pharmacologic interventions (eg, take additional antiarrhythmic medication). The efficacy of such interventions would require further dedicated study. It is notable that the patients followed in this study were already known to have a high burden of AF episodes (an average of 5.28 AF episodes per person per day). We imagine that AF episode prediction may be most useful in patients who have AF less frequently or less predictably. Additional studies would be helpful to clarify the predictive abilities of PPG tracings in patients with lower AF burdens, including those who have yet to develop AF at all.

The findings from this study also speak to the immense potential of using similar approaches to use real-time data from wearable devices for future event detection. With potential access to a high volume of nearly continuous longitudinal data from wearable devices, our ability to assess physiologic signatures immediately before a sentinel medical event has never been greater. Whether stroke, myocardial infarction, or sudden cardiac death, we think that this work highlights the potential to predict other events that have very relevant clinical significance. In addition, this may enhance our abilities to identify “pre-rhythms”: While we have some limited understanding of predisposing factors for certain rhythms (eg, long QT intervals for risk of polymorphic ventricular tachycardia), we have typically been limited in our understanding of “preventative electrophysiology” by both lack of data and lack of ability to process the data we have. Enabled by augmented prediction from wearable medical technology, we can easily envision a future where we are more effective at early detection, treatment, and prevention of episodic cardiac events.

## Funding Support and Author Disclosures

The authors have reported that they have no relationships relevant to the contents of this paper to disclose.
